# Folic Acid Supplementation Attenuates Hepatic Steatosis by Enhancing Choline Availability and Remodeling Fatty Acid Profiles in Mice Fed a High‐Fat Diet

**DOI:** 10.1096/fba.2025-00251

**Published:** 2025-10-29

**Authors:** Eva Kranenburg, Ruslan Kubant, Zeyu Yang, Adam H. Metherel, Chuck T. Chen, Jacqueline L. Beaudry, Clara E. Cho, Perlina Vaz, G. Harvey Anderson

**Affiliations:** ^1^ Department of Nutritional Sciences Temerty Faculty of Medicine, University of Toronto Toronto Ontario Canada; ^2^ Department of Human Health Sciences College of Biological Science, University of Guelph Guelph Ontario Canada; ^3^ Department of Physiology Temerty Faculty of Medicine, University of Toronto Toronto Ontario Canada

**Keywords:** choline, fatty acids, folic acid, hepatic steatosis, phosphatidylcholine, phosphatidylethanolamine, triacylglycerol

## Abstract

High‐fat diets (HFDs) are a well‐established cause of hepatic steatosis, a condition associated with altered hepatic fatty acid metabolism and reduced choline availability. Folic acid (FA) deficiency can also promote steatosis, in part by impairing choline metabolism. Although FA supplementation has been found to reduce liver fat in mice with hepatic steatosis, it is unclear if this effect is due to increased hepatic choline levels, changes in fatty acid profiles, or a combination of both. In this study, four‐week‐old male C57BL/6J mice were fed 45 kcal% HFDs with total FA content adjusted to onefold, fivefold, or tenfold AIN‐93G recommended level (2 mg/kg diet) for 15 weeks ad libitum. Hepatic triacylglycerol (TAG), choline concentrations, expression of key genes in choline metabolism, and TAG‐bound fatty acid profiles were analyzed. Mice receiving tenfold FA had lower liver weight and hepatic TAG levels compared to the onefold control group (*p* < 0.05). Both fivefold and tenfold FA supplementation increased hepatic choline concentrations and upregulated mRNA expression of choline‐metabolizing genes (*p* < 0.05), suggesting enhanced choline utilization. Additionally, tenfold FA supplementation altered the hepatic TAG fatty acid profile, reducing levels of palmitoleic acid and oleic acid (*p* < 0.05), fatty acids typically associated with *de novo* lipogenesis. A strong inverse correlation was observed between hepatic choline and TAG levels (*p* < 0.001, adjusted *R* ^2^ = 0.56), supporting a potential role for choline availability in mediating FA's protective effects. Folic acid supplementation protects against hepatic steatosis by enhancing choline availability, modulating lipid metabolism, and reducing liver fat accumulation.

## Graphical abstract

Folic acid supplementation to high‐fat diets reduced hepatic steatosis in male C57BL/6J mice, as evidenced by lower triacylglycerol (TAG) levels in the liver. These effects were associated with increased hepatic choline availability, upregulation of choline‐metabolizing genes, and alterations in the hepatic fatty acid profile, suggestive of suppressed *de novo* lipogenesis (DNL). The findings of this study highlight the potential metabolic benefits of elevated FA intake in modulating hepatic lipid homeostasis through choline‐dependent pathways.

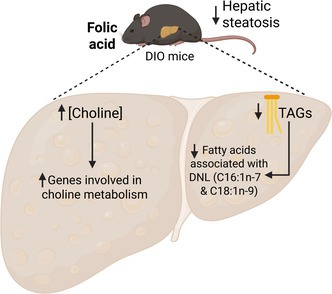

**Abbreviations**
DIO: diet‐induced obesity
FA: folic acid
FAMEs: fatty acid methyl esters
LC–MS/MS: liquid chromatography–tandem mass spectrometry
PC: phosphatidylcholine
PE: phosphatidylethanolamine
PEMT: phosphatidylethanolamine *N*‐methyltransferase
SAM: *S*‐adenosylmethionine
TAG: triacylglycerol
TLE: total lipid extract
VLDL: very low‐density lipoproteins

## 1 Introduction

Hepatic steatosis is characterized by an excessive buildup of triacylglycerol (TAG) in the liver. The global prevalence of hepatic steatosis and other metabolic dysfunction‐associated steatotic liver diseases is estimated at 30% among adults and nearly 60% in obese adults [1]. Hepatic steatosis is linked to alterations in the fatty acid composition within the liver TAGs, such as increased saturated fatty acids and decreased mono‐ and polyunsaturated fatty acids [2, 3]. It is also strongly associated with other health issues related to diet‐induced obesity (DIO), including insulin resistance [4] and impaired one‐carbon metabolism [5].

Methyl donors in the one‐carbon cycle, such as folate and choline, have been shown to influence the development of hepatic steatosis [5]. Low dietary intake of choline is linked to hepatic steatosis because it leads to a decrease in hepatic phosphatidylcholine (PC) levels, an important phospholipid for lipid metabolism and lipoprotein assembly [5]. Furthermore, multiple studies indicate that lower serum folate levels are linked to increased severity of hepatic steatosis disease [6, 7]. Another study indicated that individuals with hepatic steatosis disease frequently had lower intakes of folate from both dietary sources and supplements [8]. Reduced dietary folate availability can contribute to hepatic steatosis development and diminish choline and PC concentrations, as choline functions as an alternate methyl donor (via betaine) within the one‐carbon cycle [9]. Supplementing with folic acid (FA) in DIO rodent models has been demonstrated to reduce the development and progression of hepatic steatosis [10]; however, the underlying mechanisms are not fully understood.

Folic acid and choline interact within the one‐carbon metabolism pathway to influence metabolic processes, a relationship supported by our previous research [11]. As methyl donors, both choline and folate contribute to the production of *S*‐adenosylmethionine (SAM), a universal methyl donor [5]. We previously reported that obese C57BL/6J mice supplemented with tenfold FA had higher hepatic SAM concentrations [12]. SAM supplies methyl groups for various methylation reactions through several pathways, including the phosphatidylethanolamine *N*‐methyltransferase (PEMT) pathway [13]. This pathway, which requires three molecules of SAM to convert phosphatidylethanolamine (PE) into PC, acts as a link between choline metabolism and 1C metabolism. Reduced expression and activity of PEMT result in TAG accumulation in the liver, accompanied by a lower hepatic PC concentration [14].

Since low folate intake has been linked to decreased hepatic choline and PC concentrations, as well as the development of hepatic steatosis, this study aimed to determine whether FA supplementation to high‐fat diets (HFD) reduces hepatic steatosis through changes in hepatic choline and fatty acid profiles. We hypothesized that FA supplementation decreases hepatic steatosis by increasing choline availability and remodeling the fatty acid profile of TAGs, thereby increasing TAG clearance and lowering concentrations of hepatic fatty acids of TAGs associated with hepatic steatosis.

## 2 Materials and Methods

### 2.1 Study Design

All animal procedures were approved by the Institutional Animal Care and Use Committee at the University of Toronto (Protocol No. 20012871). Male C57BL/6J mice (Stock No: 000664) were purchased at three weeks of age from Jackson Laboratory (Bar Harbor, ME, USA) and housed in ventilated plastic cages in groups of four under a 14:10‐h light–dark cycle (lights on at 06:00, at 23°C ± 3°C) with food and water available ad libitum. Mice were acclimated for one week on a 45 kcal% HFD (primarily lard; Research Diets, Cat. No. D12451) prior to being randomized into one of three groups (*n* = 12/group) for 15 weeks: (1) HFD with onefold AIN‐93G levels of folic acid (1FA‐HFD; control), (2) HFD with fivefold AIN‐93G levels of folic acid (5FA‐HFD), or (3) HFD with tenfold AIN‐93G levels of folic acid (10FA‐HFD). Study design and diet compositions are detailed in Supporting Information (Figure S1 and Table S1, respectively).

To model hepatic steatosis in mice, we fed them HFDs for fifteen weeks. Feeding rodents diets containing at least 30 kcal% fat has been shown to promote the development of obesity and hepatic steatosis [15]. A low‐fat control group was not included in this study because the primary objective was to evaluate the dose‐dependent effects of folic acid supplementation on hepatic steatosis in the context of sustained high‐fat feeding. The 1× FA HFD group served as the control for FA supplementation, providing a baseline to assess metabolic improvements specifically attributable to FA under nutritionally stressed conditions. This design prioritizes internal consistency and translational relevance, allowing for a focused evaluation of FA as a potential intervention in obesity‐related metabolic dysfunction without introducing additional dietary variables that could confound interpretation of FA‐specific effects.

After 15 weeks on diets, all mice were euthanized by cardiac puncture under isoflurane for blood and tissue collection. Blood was collected in lithium heparin‐coated tubes (Sarstedt; Cat. No. 41.1503.105) and plasma was separated before being stored at −80°C. The liver was excised from the mouse, with a fresh portion reserved for histological analysis. The remaining tissue was flash‐frozen and stored at −80°C until further analysis.

### 2.2 Plasma TAG Concentration

Plasma TAG concentrations were quantified using the triglyceride assay (Roche Diagnostics; Cat. No. 0517407‐190), as per the manufacturer's instructions.

### 2.3 Histological Staining

Liver tissues were collected and fixed in 10% neutral buffered formalin and dehydrated in 70% ethanol until further processing. Samples were then sent to the Pathology Core at the Centre for Phenogenomics (Toronto, ON, Canada) for hematoxylin and eosin (H&E) staining and imaging.

### 2.4 Quantification of Hepatic TAG, PC, and PE

Pulverized liver samples (~50 mg) were homogenized in 6 mL of 2:1 chloroform:methanol solution (v/v) with 1.75 mL of 0.88% aqueous potassium chloride solution (w/v) and stored at 4°C for 72 h. Samples were then centrifuged for phase separation, with the lower chloroform phase (containing dissolved lipids) being collected as total lipid extract (TLE) [16].

The hepatic TAG lipid fraction was isolated by thin‐layer chromatography using G‐plates (Analtech; Cat. No. P01011). Aliquots of TLEs (representing ~5 mg liver) were loaded onto the plates along with identification standards. Lipid pools were resolved in the developing chamber containing 60:40:2 heptane:diethyl ether:glacial acetic acid (by volume). 0.1% (w/v) 8‐anilino‐1‐naphthalenesulfonic acid was then sprayed to visualize the TAG fraction under ultraviolet light, and the silica was collected for transmethylation.

For PC and PE, aliquots of TLE (representing ~10 mg liver) were loaded onto an H‐plate with Gypsum (Analtech; Cat. No. P10011) in a solution of 30:9:25:6:18 chloroform:methanol:2‐propanol:0.25% KCl:triethylamine (by volume). PC and PE were collected for transmethylation.

The silica was methylated in the presence of 6 μg of heptadecanoic acid internal standard (C17:0; Nu‐Check Prep, Item No. N‐17‐A), and fatty acid methyl esters (FAMEs) were quantified by gas chromatography‐flame ionization detection using a Varian 430 GC‐FID (SCION Instruments) equipped with a DB‐FFAP 30 m × 0.25 mm i.d. × 0.25 μm film thickness, nitroterephthalic acid modified, polyethylene glycol, capillary column (Agilent Technologies; Item No. 122‐3232). FAME peaks were identified by comparison to the retention time of the GLC‐569 external reference standard (Nu‐Check Prep) in CompassCDS version 3.0.

### 2.5 Hepatic Choline and Betaine Concentrations

Hepatic choline and betaine concentrations were measured by liquid chromatography with tandem mass spectrometry (LC–MS/MS) at the Center of Metabolomics, Baylor Scott & White Research Institute, Dallas, TX, USA, as described elsewhere [17].

### 2.6 Relative Hepatic mRNA Expression

RNA was extracted from liver tissues and homogenized using TRIzol reagent (Invitrogen, Grand Island, NY, USA), followed by chloroform extraction. The extracted RNA was then treated with 1 μg of DNase I as per the manufacturer's instructions (Thermo Fisher Scientific; Cat. No. EN0521). RNA quantification was performed using a NanoDrop 2000 Spectrophotometer (Thermo Fisher Scientific), and 500 ng was subsequently used for cDNA synthesis employing the High‐Capacity cDNA Reverse Transcription Kit (Applied Biosystems; Cat. No. 4368814) on a VeritiPro 96‐Well Thermal Cycler (Thermo Fisher Scientific). An equivalent volume of cDNA was then added to TaqMan Master Mix (Thermo Fisher Scientific) along with gene‐specific probes for phosphatidylethanolamine N‐methyltransferase (*Pemt*; Mm04933134_m1), phospholipase D1 (*Pld1*; Mm01289339_m1), choline dehydrogenase (*Chdh*; Mm00549261_m1), choline kinase alpha (*Chka*; Mm00442759_m1), choline‐phosphate cytidylyltransferase 1 alpha (*Pcyt1a*; Mm00447774_m1), fatty acid synthase (*Fasn*, Mm00662319_m1), stearoyl‐CoA desaturase 1 (*Scd1*, Mm00772290_m1), peroxisome proliferator‐activated receptor alpha (*Ppara*, Mm00440939_m1), and carnitine palmitoyltransferase 1A (*Cpt1a*, Mm01231183_m1), which were run on a QuantStudio 5 Real‐Time PCR System (Thermo Fisher Scientific). Candidate reference genes glyceraldehyde 3‐phosphate dehydrogenase (*Gapdh*), peptidylprolyl isomerase A (*Ppia*), TATA‐box binding protein (*Tbp*), and beta‐actin (*Actb*) were evaluated for expression stability using geNorm within qBase+, with *Gapdh* (Mm99999915_g1) being selected as the optimal reference gene for normalization. Gene expression results were expressed as fold‐change using the 2^−ΔΔCT^ (cycle threshold) method.

### 2.7 Statistical Analyses

Our primary outcome, hepatic steatosis, was defined by hepatic TAG concentrations of > 20 mg/g tissue [18]. Based on previous research [19], a minimum sample size of six mice per group was determined to be sufficient to detect a 10% difference in hepatic TAGs between groups at a significance level of *p* ≤ 0.05 and a power of 0.80 (by G Power software, version 3.1). The secondary outcome was choline concentrations in the liver. Exploratory measures included liver weight, fatty acid profile of hepatic TAGs, PE and PC concentrations and fatty acid profile in the liver, plasma TAGs, and the expression of hepatic genes involved in choline metabolism (phosphatidylethanolamine N‐methyltransferase, phospholipase D, choline dehydrogenase, choline kinase alpha, and CTP: phosphocholine cytidylyltransferase).

SAS v.9.4 (SAS Institute Inc., Cary, NC, USA) and RStudio Version 2024.12.1 were used for statistical analyses. Pairwise comparisons were performed using the Tukey–Kramer test following one‐way ANOVA, which adjusts for multiple comparisons by controlling the family‐wise error rate. Simple linear regression analysis with hepatic choline as the independent variable and liver TAG as the dependent variable was performed. The goodness of fit was assessed using the adjusted coefficient of determination (adjusted *R* ^2^), and statistical significance was determined using the *F*‐test for the overall model. Statistical significance was defined as *p* ≤ 0.05. All values are presented as mean ± standard error of mean (SEM).

## 3 Results

### 3.1 Folic Acid Supplementation Reduced Hepatic Steatosis

**FIGURE 1:**
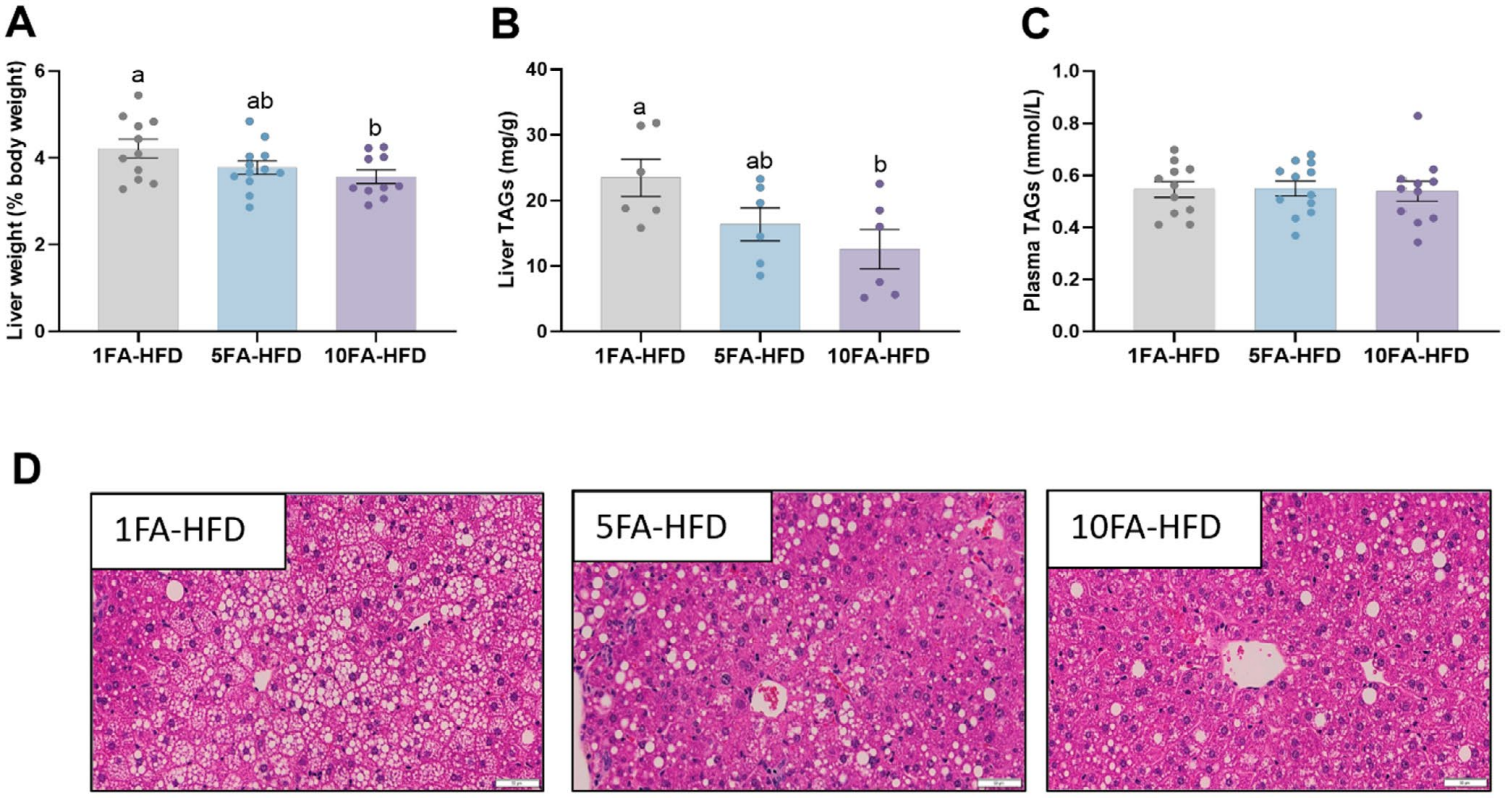
Folic acid supplementation reduced fatty liver. Tenfold folic acid high‐fat diet‐fed mice had lower liver weight adjusted for body weight (A) and liver TAGs (B), although plasma TAGs were not different (C). Reduced fat accumulation is shown in H&E‐stained liver sections (D). H&E stains (20×) presented are representative of the groups. Different letters indicate statistically significant differences between the means of groups by one‐way ANOVA with Tukey–Kramer post hoc test. Data presented as means ± SEM *n* = 11–12/group (A and C) and *n* = 6/group (B and D).

After fifteen weeks, 10FA‐HFD mice had 16% lower liver weight after adjusting for final body weight compared to the control group (1FA‐HFD) (*p* = 0.049) (Figure 1A), whereas 5FA‐HFD mice were not different from 1FA‐HFD nor 10FA‐HFD (*p* > 0.05). Moreover, mice in the 10FA‐HFD group had 46% lower liver TAG concentrations compared to the control (*p* = 0.043), while TAG concentrations in the liver of 5FA‐HFD mice did not differ from 1FA‐HFD nor 10FA‐HFD mice (*p* > 0.05) (Figure 1B). The lipid droplets in the liver were also visualized by H&E staining (Figure 1D), showing less lipid accumulation in the liver of 10FA‐HFD mice. In contrast, plasma TAG concentrations did not differ among groups (*p* > 0.05; Figure 1C).

### 3.2 Folic Acid Supplementation Modified Hepatic Fatty Acid Profiles of TAGs

FA supplementation modified the hepatic fatty acid profile of the TAGs (Table 1) with four fatty acids differing even after adjusting to total fatty acid concentrations (Table S2). Specifically, 10FA‐HFD mice had lower concentrations of saturated fatty acids (46%, *p* = 0.047), monounsaturated fatty acids (49%, *p* = 0.041), and omega‐3 polyunsaturated fatty acids (42%, *p* = 0.045) compared to the control (*p* < 0.05) (Table 1). 10FA‐HFD mice had lower concentrations of multiple fatty acids, including palmitoleic, oleic, and eicosenoic acids, along with reductions in both omega‐3 (EPA, DPA) and omega‐6 (eicosadienoic, dihomo‐gamma linolenic acid, docosadienoic, adrenic) polyunsaturated fatty acids (*p* < 0.05; Table 1). Moreover, concentrations of several fatty acids, including palmitic acid and stearic acid, decreased in fivefold and tenfold FA groups, although these effects did not reach significance (*p* = 0.054 and 0.057, respectively). Tenfold FA supplementation resulted in higher amounts of behenic acid, erucic acid, gamma linolenic acid, and n‐6 docosapentaenoic acid relative to the total fatty acid pool (*p* < 0.05), with 5FA‐HFD mice not having significant differences compared to 1FA‐HFD nor 10FA‐HFD (*p* > 0.05) (Table S2).

**TABLE 1:** Fatty acid concentrations of hepatic TAGs.

Fatty acids (μmol/g)	1FA‐HFD	5FA‐HFD	10FA‐HFD	*p*
Saturated fatty acids				
Mystiric acid (C14:0)	1.55 ± 0.28	0.91 ± 0.14	0.77 ± 0.23	0.057
Palmitic acid (C16:0)	61.91 ± 7.25	44.05 ± 6.51	33.83 ± 8.62	0.054
Stearic acid (C18:0)	4.85 ± 1.13	2.89 ± 0.38	2.21 ± 0.43	0.057
Arachidic acid (C20:0)	0.42 ± 0.07	0.30 ± 0.02	0.34 ± 0.07	0.364
Behenic acid (C22:0)	0.04 ± 0.01^ab^	0.03 ± 0.00^a^	0.05 ± 0.00^b^	0.019
Ʃ Saturated fatty acids	68.77 ± 8.29^a^	48.19 ± 7.02^ab^	37.20 ± 9.33^b^	0.047
Monounsaturated fatty acids				
Myristoleic acid (C14:1)	0.04 ± 0.02	0.01 ± 0.01	0.01 ± 0.01	0.270
Palmitoleic acid (C16:1n‐7)	9.43 ± 1.23^a^	5.58 ± 0.96^ab^	4.80 ± 1.57^b^	0.048
Oleic acid (C18:1n‐9)	105.55 ± 13.40^a^	70.10 ± 11.82^ab^	54.32 ± 14.09^b^	0.041
Vaccenic acid (C18:1n‐7)	7.80 ± 0.86	4.85 ± 1.02	3.95 ± 1.27	0.055
Eicosenoic acid (C20:1n‐9)	2.27 ± 0.28^a^	1.36 ± 0.22^ab^	1.26 ± 0.34^b^	0.045
Erucic acid (C22:1n‐9)	0.12 ± 0.03	0.07 ± 0.01	0.09 ± 0.02	0.358
Ʃ Monounsaturated fatty acids	125.20 ± 15.66^a^	81.98 ± 13.83^ab^	64.43 ± 17.26^b^	0.041
n‐3 Polyunsaturated fatty acids				
Alpha‐linolenic acid (ALA, C18:3n‐3)	1.37 ± 0.25	1.14 ± 0.16	0.86 ± 0.14	0.195
Eicosatrienoic acid (C20:3n‐3)	0.09 ± 0.02	0.05 ± 0.01	0.06 ± 0.01	0.143
Eicosapentaenoic acid (EPA, C20:5n‐3)	0.42 ± 0.03^a^	0.45 ± 0.06^a^	0.26 ± 0.05^b^	0.031
n‐3 Docosapentaenoic acid (DPA, C22:5n‐3)	0.34 ± 0.09^a^	0.27 ± 0.10^ab^	0.19 ± 0.09^b^	0.042
Docosahexaenoic acid (DHA, C22:6n‐3)	2.82 ± 0.29	2.29 ± 0.42	1.58 ± 0.25	0.052
Ʃ n‐3 Polyunsaturated fatty acids	5.73 ± 0.51^a^	4.76 ± 0.76^ab^	3.33 ± 0.56^b^	0.045
n‐6 Polyunsaturated fatty acids				
Linoleic acid (C18:2n‐6)	37.90 ± 5.57	30.71 ± 4.89	21.81 ± 3.87	0.094
Gamma linolenic acid (C18:3n‐6)	0.71 ± 0.07	0.71 ± 0.08	0.51 ± 0.08	0.124
Eicosadienoic acid (C20:2n‐6)	0.76 ± 0.12^a^	0.46 ± 0.06^ab^	0.39 ± 0.09^b^	0.030
Dihomo‐gamma linolenic acid (C20:3n‐6)	1.81 ± 0.22^a^	1.22 ± 0.15^ab^	0.98 ± 0.23^b^	0.031
Arachidonic acid (ARA C20:4n‐6)	3.38 ± 0.35^a^	2.82 ± 0.41^ab^	2.11 ± 0.34^b^	0.018
Docosadienoic acid (C22:2n‐6)	0.81 ± 0.09^a^	0.41 ± 0.09^b^	0.45 ± 0.08^b^	0.012
Adrenic acid (C22:4n‐6)	1.06 ± 0.13^a^	0.67 ± 0.09^ab^	0.57 ± 0.11^b^	0.018
n‐6 Docosapentaenoic acid (C22:5n‐6)	0.43 ± 0.05	0.30 ± 0.04	0.29 ± 0.05	0.088
Ʃ n‐6 polyunsaturated fatty acids	46.86 ± 6.07	37.31 ± 5.67	27.12 ± 4.80	0.072

*Note:* Different superscript letters indicate statistically significant differences between the means of groups by one‐way ANOVA with Tukey–Kramer post hoc test. Data presented as means ± SEM *n* = 6/group.

Abbreviations: 1FA‐HFD, onefold folic acid‐high fat diet; 5FA‐HFD, fivefold folic acid‐high fat diet; 10FA‐HFD, tenfold folic acid‐high fat diet; TAGs, triacylglycerol.

### 3.3 Folic Acid Supplementation Increased Hepatic Choline Concentrations

**FIGURE 2:**
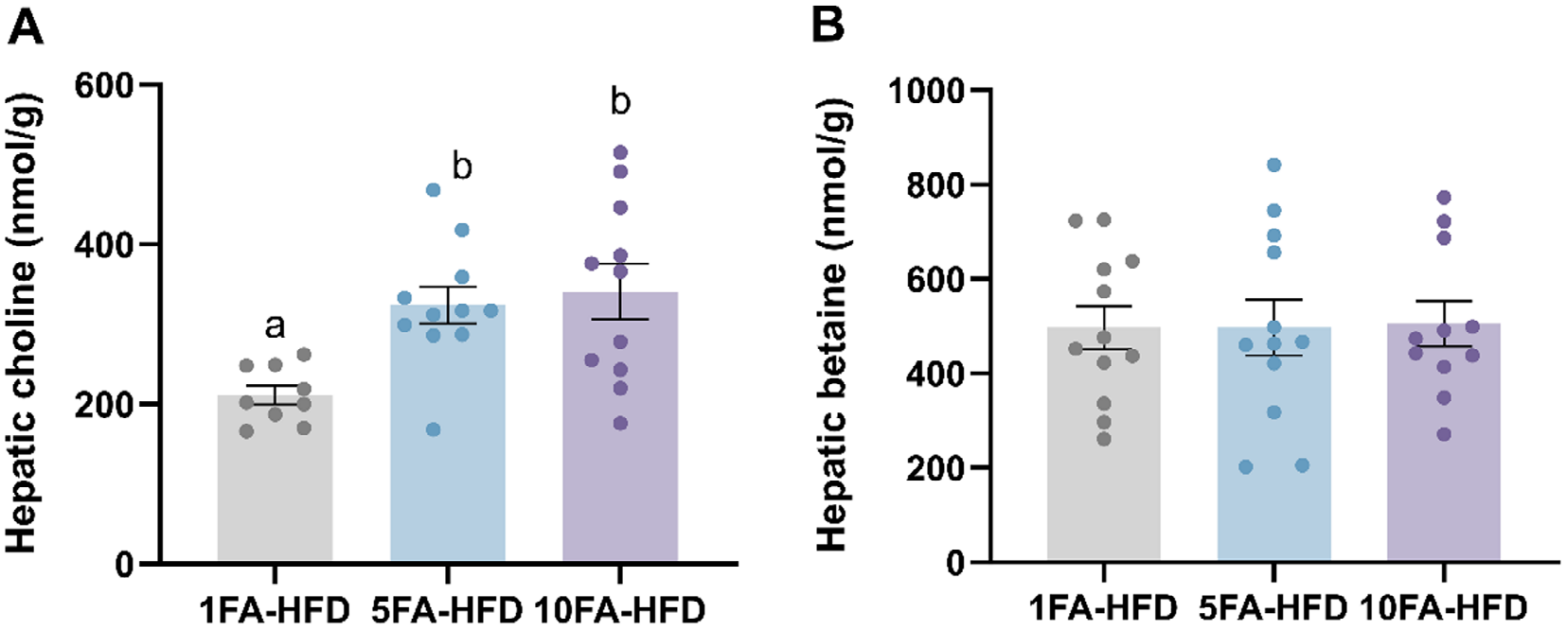
Hepatic choline (A) and betaine (B) concentrations. Different letters indicate statistically significant differences between the means of groups by one‐way ANOVA with Tukey–Kramer post hoc test. Data presented as means ± SEM *n* = 9–11/group (A) and *n* = 11–12/group (B).

**FIGURE 3:**
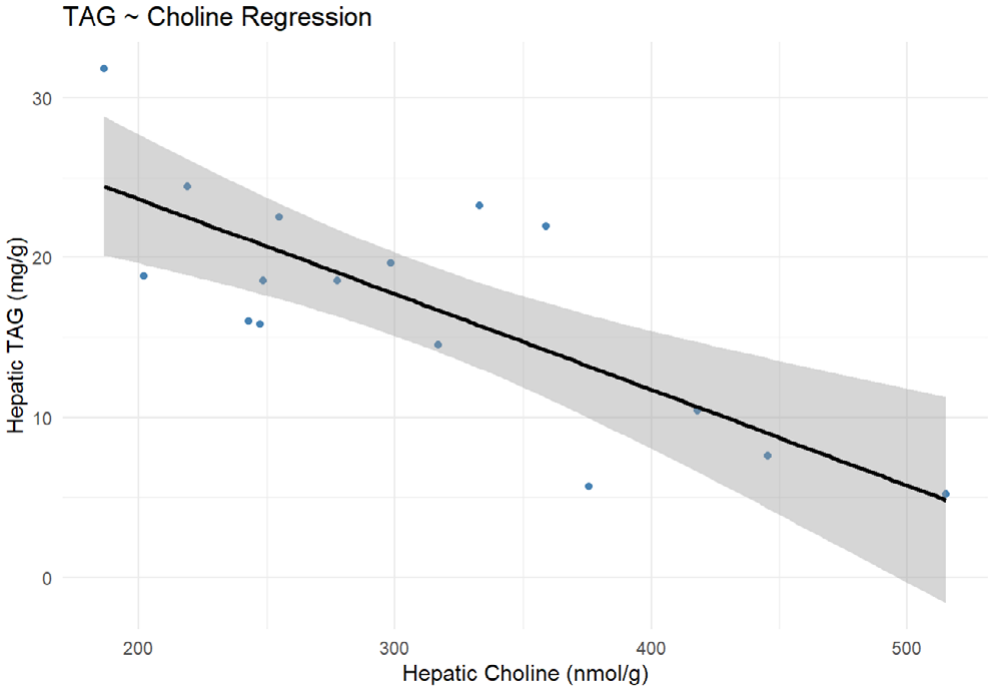
Inverse relationship between hepatic choline concentration and liver TAG levels. Linear regression analysis shows that hepatic choline predicts liver TAG (adjusted *R* ^2^ = 0.56, *p* < 0.001). Data points represent individual samples; the solid line is the regression fit with a 95% confidence interval shaded. *n* = 18.

Hepatic choline concentrations were 53% higher in 5FA‐HFD (*p* = 0.016) and 61% higher in 10FA‐HFD (*p* = 0.0054) mice compared to the control (Figure 2A). In contrast, hepatic betaine concentrations did not differ between groups (*p* > 0.05; Figure 2B). A significant inverse relationship was observed between hepatic TAG and choline concentrations in the liver (adjusted *R* ^2^ = 0.56; *p* = 0.0005; Figure 3).

### 3.4 Folic Acid Supplementation Increased Hepatic Phosphatidylethanolamine but Not Phosphatidylcholine Levels

**FIGURE 4:**
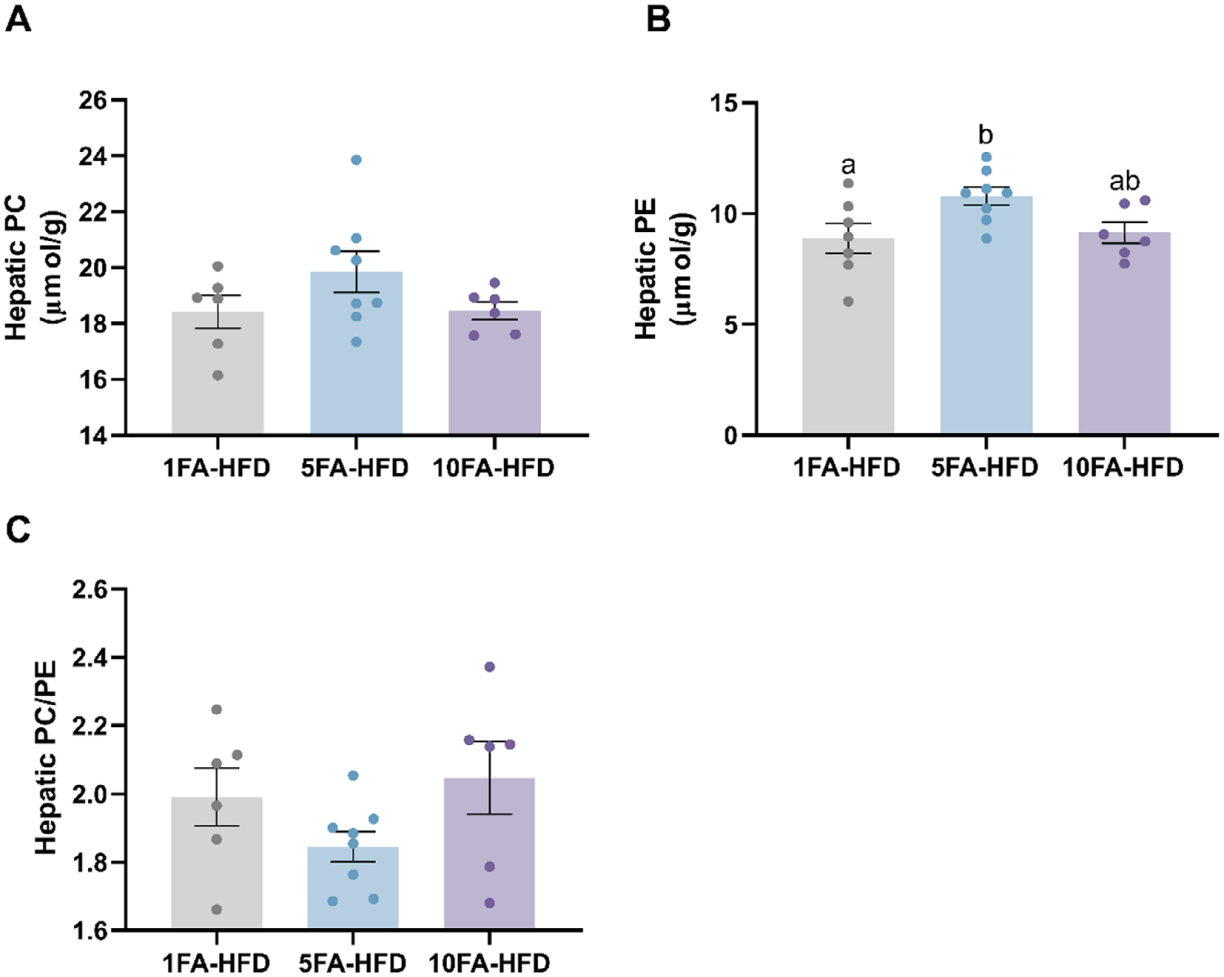
Hepatic phosphatidylcholine (A) and phosphatidylethanolamine (B) concentrations and their ratio (C). Different letters indicate statistically significant differences between the means of groups by one‐way ANOVA with Tukey–Kramer post hoc test. Data presented as means ± SEM *n* = 6–8/group.

The observation of increased choline levels in the liver prompted us to investigate whether this change was linked to alterations in hepatic PC and PE concentrations. Specifically, we were interested in understanding if FA‐mediated increases in hepatic choline influenced the ratio of PC to PE, a key factor in liver health [20]. Concentrations of PC in the liver did not differ between groups (*p* > 0.05; Figure 4A). However, hepatic PE levels were higher in 5FA‐HFD mice compared to the control (*p* = 0.042; Figure 4B). Hepatic PE concentrations did not differ between 10FA‐HFD and the control, nor between 10FA‐HFD and 5FA‐HFD mice (*p* > 0.05). The PC/PE ratio was not different across groups (*p* > 0.05; Figure 4C).

The fatty acid profile of PC was not affected by treatment (*p* > 0.05; Table S3). When normalized to total fatty acids, linoleic acid in the PC phospholipid fraction was higher in 10FA‐HFD mice compared to the control (*p* < 0.05; Table S4). In the PE phospholipid fraction, concentrations of n‐3 docosapentaenoic acid (C22:5n‐3) were higher in 5FA‐HFD mice compared to the control, while docosahexaenoic acid (DHA, C22:6n‐3) was lower in 10FA‐HFD mice compared to the 5FA‐HFD group (*p* < 0.05; Table 2). Moreover, arachidonic acid (C20:4n‐6) was lower in 10FA‐HFD mice compared to 5FA‐HFD (*p* < 0.05) and intermediate to the control (*p* > 0.05). The concentrations of total omega‐3 polyunsaturated fatty acids were higher in 5FA‐HFD compared to both 1FA‐HFD and 10FA‐HFD groups (*p* = 0.01). Upon normalization to total fatty acids, docosahexaenoic acid was significantly lower in 10FA‐HFD mice compared to 5FA‐HFD (*p* < 0.05). Interestingly, when normalized to total fatty acids, oleic acid was lower in 5FA‐HFD mice compared to control (*p* < 0.05), with intermediate effects in 10FA‐HFD mice (*p* > 0.05; Table S5). Pentadecanoic acid (C15:0) was higher in 10FA‐HFD mice compared to 5FA‐HFD mice (*p* < 0.05), while linoleic acid (C18:2n‐6) was higher in the 10FA‐HFD group compared to the control (*p* < 0.05) (Table S5). Monounsaturated fatty acids (*p* < 0.05) and total n‐3 polyunsaturated fatty acids were higher in the 5FA‐HFD group compared to the control (*p* < 0.01; Table S5).

**TABLE 2:** Fatty acid concentrations of hepatic PE.

Fatty acids (μmol/g)	1FA‐HFD	5FA‐HFD	10FA‐HFD	*p*
Saturated fatty acids				
Lauric acid (C12:0)	0.01 ± 0.00	0.00 ± 0.00	0.01 ± 0.00	0.076
Mystiric acid (C14:0)	0.03 ± 0.00	0.03 ± 0.00	0.04 ± 0.00	0.119
Pentadecanoic acid (C15:0)	0.01 ± 0.00	0.01 ± 0.00	0.01 ± 0.00	0.265
Palmitic acid (C16:0)	3.66 ± 0.16	3.82 ± 0.15	3.59 ± 0.16	0.555
Stearic acid (C18:0)	3.33 ± 0.17	3.80 ± 0.16	3.26 ± 0.17	0.063
Arachidic acid (C20:0)	0.03 ± 0.00	0.04 ± 0.00	0.04 ± 0.00	0.267
Behenic acid (C22:0)	0.01 ± 0.00	0.01 ± 0.00	0.01 ± 0.00	0.867
Lignoceric acid (C24:0)	0.01 ± 0.00	0.01 ± 0.00	0.01 ± 0.00	0.479
Ʃ Saturated fatty acids	7.08 ± 0.31	7.72 ± 0.29	6.97 ± 0.31	0.185
Monounsaturated fatty acids				
Myristoleic acid (C14:1)	0.00 ± 0.00	0.00 ± 0.00	0.01 ± 0.00	0.577
Palmitoleic acid (C16:1n‐7)	0.06 ± 0.01	0.06 ± 0.01	0.06 ± 0.01	0.863
Sapienic acid (C16:1n‐9)	0.01 ± 0.00	0.01 ± 0.00	0.01 ± 0.00	0.726
Vaccenic acid (C18:1n‐7)	0.23 ± 0.02	0.24 ± 0.02	0.19 ± 0.02	0.145
Oleic acid (C18:1n‐9)	2.63 ± 0.12	2.74 ± 0.11	2.54 ± 0.12	0.453
Gondoic acid (C20:1n‐9)	0.05 ± 0.00	0.06 ± 0.00	0.05 ± 0.00	0.560
Erucic acid (C22:1n‐9)	0.04 ± 0.00	0.05 ± 0.00	0.04 ± 0.00	0.557
Nervonic acid (C24:1n‐9)	0.01 ± 0.00	0.01 ± 0.00	0.01 ± 0.00	0.149
Ʃ Monounsaturated fatty acids	3.03 ± 0.14	3.16 ± 0.13	2.91 ± 0.14	0.414
n‐3 Polyunsaturated fatty acids				
α‐linolenic acid (ALA, C18:3n‐3)	0.01 ± 0.00	0.01 ± 0.00	0.01 ± 0.00	0.302
Eicosatrienoic acid (ETE, C20:3n‐3)	0.01 ± 0.00	0.01 ± 0.00	0.01 ± 0.00	0.328
Eicosapentaenoic acid (EPA, C20:5n‐3)	0.03 ± 0.00	0.04 ± 0.00	0.03 ± 0.00	0.093
n‐3 Docosapentaenoic acid (DPA, C22:5n‐3)	0.08 ± 0.01^a^	0.11 ± 0.01^b^	0.08 ± 0.01^ab^	0.039
Docosahexaenoic acid (DHA, C22:6n‐3)	3.73 ± 0.38^a^	5.17 ± 0.35^b^	3.53 ± 0.38^a^	0.010
Ʃ n‐3 polyunsaturated fatty acids	3.85 ± 0.39^a^	5.33 ± 0.36^b^	3.65 ± 0.39^a^	0.010
n‐6 Polyunsaturated fatty acids				
Linoleic acid (C18:2n‐6)	0.76 ± 0.06	0.87 ± 0.06	0.86 ± 0.06	0.363
γ‐Linolenic acid (C18:3n‐6)	0.00 ± 0.00	0.01 ± 0.00	0.01 ± 0.00	0.776
Eicosadienoic acid (C20:2n‐6)	0.03 ± 0.00	0.03 ± 0.00	0.03 ± 0.00	0.580
Dihomo‐γ‐linolenic acid (C20:3n‐6)	0.11 ± 0.01	0.13 ± 0.01	0.10 ± 0.01	0.090
Arachidonic acid (ARA, C20:4n‐6)	3.71 ± 0.22^ab^	4.47 ± 0.20^a^	3.60 ± 0.22^b^	0.019
Adrenic acid (C22:4n‐6)	0.07 ± 0.01	0.08 ± 0.01	0.07 ± 0.01	0.243
n‐6 Docosapentaenoic acid (C22:5n‐6)	0.08 ± 0.01	0.10 ± 0.01	0.09 ± 0.01	0.390
Ʃ n‐6 polyunsaturated fatty acids	4.76 ± 0.26^a^	5.89 ± 0.26^b^	4.76 ± 0.26^a^	0.010

*Note:* Different superscript letters indicate statistically significant differences between the means of groups by one‐way ANOVA with Tukey–Kramer post hoc test. Data presented as means ± SEM *n* = 6–7/group.

Abbreviations: 1FA‐HFD, onefold folic acid‐high fat diet; 5FA‐HFD, fivefold folic acid‐high fat diet; 10FA‐HFD, tenfold folic acid‐high fat diet; PE, phosphatidylethanolamine.

### 3.5 Folic Acid Modified Expression of Genes Involved in Hepatic Choline Metabolism

**FIGURE 5:**
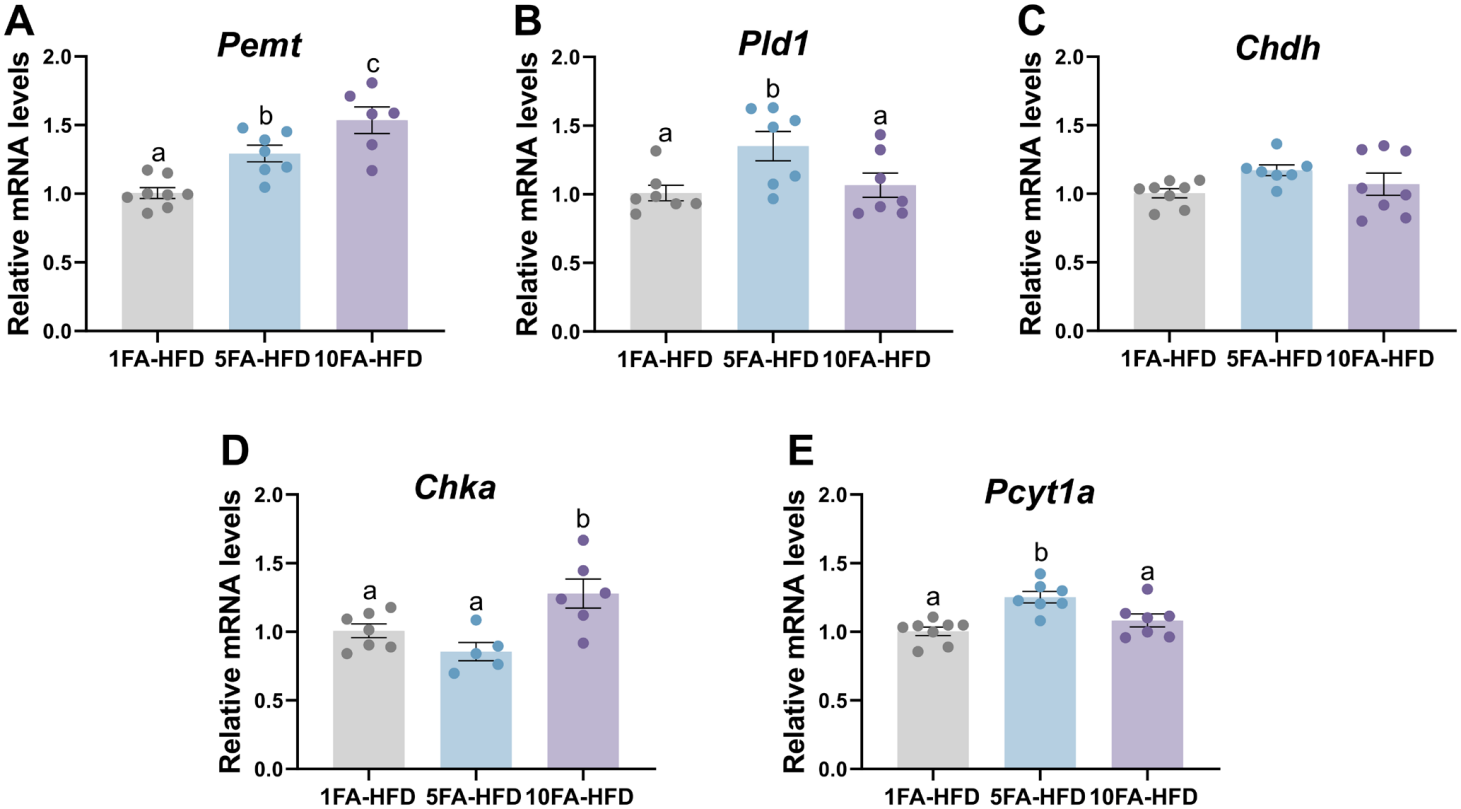
Relative mRNA expression levels of (A) phosphatidylethanolamine N‐methyltransferase (*Pemt*), (B) phospholipase D1 (*Pld1*), (C) choline dehydrogenase (*Chdh*), (D) choline kinase a (*Chka*), and (E) choline‐phosphate cytidylyltransferase A (*Pcyt1a*) in the liver. Different letters indicate statistically significant differences between the means of groups by one‐way ANOVA with Tukey–Kramer post hoc test. Data presented as means ± SEM, *n* = 5–8/group.

To understand the differences in hepatic choline metabolism and how they relate to gene variations, we measured the mRNA expression of genes involved in the choline pathway, particularly phosphatidylethanolamine N‐methyltransferase (*Pemt*), phospholipase D1 (*Pld1*), choline dehydrogenase (*Chdh*), choline kinase alpha (*Chka*), and phosphate cytidylyltransferase 1A (*Pcyt1a*). The expression of *Pemt* was 28% and 47% higher in 5FA‐HFD and 10FA‐HFD mice compared to the control, respectively (*p* = 0.018 and *p* = 0.0002; Figure 5A). Expression levels of *Pemt* were 24% higher in 10FA‐HFD mice compared to 5FA‐HFD mice (*p* < 0.05). *Pld1* expression was 34% higher in 5FA‐HFD mice compared to the control (*p* = 0.030; Figure 5B) and 29% higher than 10FA‐HFD mice, whereas 10FA‐HFD mice did not differ compared to the control (*p* > 0.05). mRNA levels of *Chdh* were not different between groups (*p* > 0.05; Figure 5C).

Within the CDP‐choline pathway, genes encoding rate‐limiting enzymes, choline kinase and choline‐phosphate cytidylyltransferase A, were measured in the liver. *Chka* expression was 27% higher in 10FA‐HFD mice compared to the control (*p* = 0.05) and 42% higher than 5FA‐HFD (*p* = 0.006; Figure 5D). *Pcyt1a* expression in 5FA‐HFD mice was 25% higher than the control (*p* = 0.0007) and 17% higher than 10FA‐HFD (*p* = 0.02) mice (Figure 5E).

## 4 Discussion

Previously, we showed that FA supplementation to a HFD reduces body weight, adiposity, and insulin resistance in male mice by modulating the one‐carbon cycle and methylation pathways [15]. Here, we extend these findings by investigating FA's impact on choline‐linked lipid metabolism. We demonstrate that FA supplementation attenuates hepatic steatosis and improves the hepatic TAG fatty acid profile during sustained high‐fat feeding, even without reducing dietary fat. These protective effects are associated with increased hepatic choline availability, supporting its role as a methyl donor in PC synthesis and VLDL‐mediated lipid export. Importantly, benefits occur in a metabolically challenged state, suggesting FA may serve as a supportive intervention for individuals consuming HFDs with early‐stage fatty liver disease.

FA supplementation (10‐97‐fold AIN‐93G doses in rodents) has been shown to reduce hepatic steatosis, and this effect is believed to be mediated through several mechanisms, including improved hepatic lipid metabolism, reduced oxidative stress, and modulation of inflammatory pathways [19, 21, 22]. Similarly, we demonstrate that tenfold FA additions resulted in lower liver weights after adjusting for final body weights. Further supporting this, mice fed with tenfold FA had approximately 50% lower liver TAGs than the control group, as visualized by H&E staining. Control mice met the criteria for hepatic steatosis (> 20 mg TAG/g liver) [18], and the reduction observed in tenfold FA‐supplemented mice indicates that FA alleviated hepatic steatosis. However, the lower liver TAG concentrations in mice consuming tenfold FA did not result in changes in plasma TAG levels. This contrasts with other reports that showed reduced plasma TAG concentrations in response to FA supplementation in Wistar rats [19, 23], likely due to species‐specific differences, as high‐fat feeding (45 and 60 kcal% fat) in C57BL/6J mice has been reported to not affect plasma TAG levels [24, 25]. While plasma TAG levels did not decrease in our model, likely due to species‐specific metabolic responses, hepatic outcomes provide a more direct indicator of steatosis.

The liver‐to‐body weight ratio in our HFD control group (~4.5%) aligns well with established models of diet‐induced hepatic steatosis, where ratios typically rise from a normal range of 2.0%–2.5% in lean mice to 4.0%–5.0% under prolonged HFD feeding [19]. This increase reflects both hepatomegaly and lipid accumulation, supporting the metabolic relevance of our model. Hepatic steatosis was assessed using H&E staining, which revealed macrovesicular vacuolization consistent with lipid droplet formation. This finding was further confirmed by quantitative measurement of hepatic TAG content, a more objective and reproducible indicator of fat accumulation than semiquantitative histology alone. While Oil Red O staining provides enhanced specificity for neutral lipids and would have strengthened the morphological assessment, it was not performed in this study. We acknowledge this as a limitation. However, given that TAG quantification offers a direct biochemical measure of liver fat, our conclusion of reduced steatosis in the 10× FA group is well‐supported.

Given that reduced steatosis could potentially result from decreased food intake, it is important to note that caloric consumption did not differ across groups, as previously reported in this cohort [12], indicating that the observed improvements in liver fat are independent of energy intake. Despite this, mice receiving 10× folic acid exhibited lower final body weight and reduced white adipose tissue mass compared to the 1× control group, suggesting broader improvements in lipid or energy metabolism. The absence of differences in food intake further indicates that the dose‐dependent reductions in hepatic alpha‐linolenic and linoleic acid levels are not due to altered dietary intake, but likely reflect changes in hepatic fatty acid handling, distribution, or metabolism. These shifts may be linked to FA's role in one‐carbon metabolism and PC synthesis, processes that influence membrane lipid composition and fatty acid partitioning. Together, these findings support a direct metabolic effect of FA supplementation under high‐fat feeding, independent of changes in food consumption. Given that the metabolic improvements observed were independent of food intake, we next examined the hepatic TAG fatty acid composition to gain insight into how FA supplementation influences lipid remodeling.

For the first time, we report the hepatic fatty acid profile of TAGs in response to FA supplementation. Total saturated, n‐3 polyunsaturated, and monounsaturated fatty acids were lower in mice supplemented with tenfold FA compared to the control. Among the affected fatty acids were palmitoleic acid (C16:1n‐7), a marker of *de novo* lipogenesis (DNL), and oleic acid (C18:1n‐9), a major component of TAGs. These fatty acids are commonly elevated in hepatic steatosis [26, 27], and their reduction following FA supplementation suggests that FA may attenuate hepatic lipid accumulation and reduce hepatic steatosis severity. Since most fatty acids decreased in response to FA supplementation, this may reflect reduced hepatic fatty acid influx, increased oxidation of fatty acids, or lower retention of TAGs due to improved lipid turnover.

Adding FA to a HFD resulted in higher levels of choline in the liver but did not change the levels of hepatic betaine. The lack of change in betaine and *Chdh* mRNA expression, despite increased choline availability, may suggest a shift in how choline is metabolized. Previous studies show that folate deficiency increases *Chdh* expression [28], indicating that folate status affects choline utilization. When FA is supplemented, choline may be redirected away from breakdown via the CHDH pathway and instead used in alternative pathways—such as PEMT and the CDP‐choline pathway. This metabolic shift could help increase the production of PC, a key component for cell membrane structure and for packaging fats into very‐low‐density lipoprotein (VLDL) particles, which transport fat out of the liver [29].

There was a significant inverse relationship between hepatic TAG and choline concentrations, suggesting that reductions in liver TAGs can partly be explained by higher hepatic choline content. This aligns with the literature supporting a protective role of choline in the development and progression of hepatic steatosis, primarily proposed to be mediated through higher PC concentrations [28]. Since choline can be obtained from two sources—dietary intake and the catabolism of PC—we examined whether the increased hepatic choline content could be due to changes in PC levels. PC is synthesized via two pathways: CDP‐choline and PEMT. While the CDP‐choline pathway primarily regulates PC biosynthesis, approximately 30% of PC is produced from PE via the PEMT pathway, which requires three molecules of SAM [29]. Recently, we reported that tenfold FA supplementation to the HFD of mice resulted in higher hepatic SAM concentrations [12], implying increased availability of SAM for the PEMT pathway. In the liver, *Pemt* expression levels were higher in the FA‐supplemented groups. Despite increased *Pemt* gene expression, PC levels remained unchanged, possibly because the overall metabolic flux is balanced by other pathways, such as the CDP‐choline pathway, which accounts for around 70% of PC biosynthesis in the liver [29]. FA supplementation increased the expression of genes encoding rate‐limiting enzymes in the CDP‐choline pathway, as well as phospholipase D1, involved in PC catabolism, which may indicate higher turnover of PC and could partially explain the elevated hepatic choline concentrations. Fivefold FA led to more dynamic changes in genes encoding key enzymes in choline metabolism, with changes in the CDP‐choline and choline catabolism pathways; whereas tenfold FA supplementation had more pronounced effects on the PEMT pathway. This may suggest a shift in how choline is metabolized, dependent on FA's effects on 1C metabolism and substrate availability. Although further research is needed to clarify the exact mechanisms, these results suggest that folate influences various choline pathways to regulate its concentrations in the liver.

Surprisingly, fivefold FA supplementation resulted in higher PE concentrations in the liver, whereas tenfold FA‐fed mice had levels similar to the control. Given the lack of change in hepatic PC levels and no increase in hepatic SAM concentrations in the studied mice [12], it is possible that insufficient SAM availability may have constrained PEMT activity, which requires SAM as a methyl donor [30]. In contrast, mice fed tenfold FA exhibited higher *Pemt* expression despite no differences in hepatic PE or PC levels compared to controls. However, static metabolite measurements may not reflect the rate at which metabolites are utilized within a pathway [31]. Since these mice showed lower TAG levels and higher concentrations of SAM in the liver [12], the unchanged PC concentrations could reflect increased turnover rather than synthesis rate [32]—suggesting enhanced conversion of PE to PC via PEMT, supporting increased VLDL secretion, a process not evident from steady‐state PC levels alone. Increased VLDL secretion can help clear excess fat (TAGs) from the liver, thereby reducing hepatic steatosis [28]. Moreover, the fatty acid profiles of PE changed in response to FA supplementation. Mice supplemented with tenfold FA showed higher levels of pentadecanoic acid (C15:0), an odd‐chain saturated fatty acid linked in studies to lower inflammation and reduced risk of hepatic steatosis [28], suggesting a potential metabolic benefit of high‐dose FA supplementation. FA supplementation also reduced hepatic arachidonic acid (C20:4n‐6) levels, which may contribute to the improvement in hepatic steatosis. Arachidonic acid is a pro‐inflammatory omega‐6 fatty acid and a precursor to eicosanoids that promote inflammation and insulin resistance—key drivers of hepatic steatosis disease progression [33]. Given that FA supplementation has been shown to reduce hepatic inflammation in mice [21], the observed decrease in hepatic steatosis severity may be partly due to lower levels of pro‐inflammatory fatty acids like arachidonic acid, supporting an anti‐inflammatory role for FA in liver metabolism.

In addition to its effects on hepatic lipid composition and inflammation, FA supplementation also exerts significant metabolic benefits beyond the liver. A ~50% reduction in the homeostatic model of assessment for insulin resistance (HOMA‐IR) with FA supplementation at both 5× and 10× doses was previously reported in our cohort [12], indicating improved systemic insulin sensitivity, which primarily reflects hepatic insulin action under fasting conditions [34]. Given the strong link between hepatic insulin resistance and steatosis [35], this improvement may contribute to the overall metabolic benefits observed. However, the significant reduction in hepatic TAGs and liver weight was only evident in the tenfold group, despite similar HOMA‐IR improvements at both doses. This dissociation suggests that enhanced insulin sensitivity, while beneficial, is not the primary driver of the anti‐steatotic effects. Moreover, insulin signaling, which influences DNL activity, may have been improved, as suggested by reduced HOMA‐IR. However, this did not translate into detectable changes in hepatic lipogenic transcripts, *Fasn* and *Scd1* (Figure S2), despite reductions in liver TAG content and lower levels of their end‐product fatty acids, such as palmitoleic and oleic acid. This apparent disconnect suggests that transcriptional regulation of canonical DNL pathways is also unlikely to be the primary driver of improved steatosis with FA supplementation. However, we cannot rule out alterations in DNL flux at the metabolic level, as enzyme activity may be modulated posttranslationally or by substrate availability (e.g., acetyl‐CoA, NADPH) independently of gene expression. We also did not assess serum lipoprotein levels, including VLDL, which limits our ability to evaluate hepatic lipid export. Given that PC, a product of choline metabolism, is essential for VLDL assembly and secretion, enhanced lipid efflux from the liver could contribute to reduced fat accumulation, even in the absence of altered DNL gene expression. These possibilities highlight the complexity of hepatic lipid handling and underscore the need for future studies using isotopic tracers or direct measures of VLDL secretion to clarify the mechanistic role of FA in liver fat metabolism.

A major strength of this study is that it is the first to examine how FA supplementation affects liver choline metabolism and fatty acid concentrations in the setting of hepatic steatosis, using a highly controlled dietary approach. All diets provided the same number of calories and had identical macronutrient and fatty acid compositions, differing only in the amount of supplemental FA. Because dietary choline was kept constant across groups, this design reduces the influence of confounding variables and allows for a clear evaluation of FA's specific effects. It also enables us to assess whether improvements in hepatic steatosis are linked to changes in how choline is used in the liver.

A key limitation of this study is the inability to track the real‐time flux of choline through metabolic pathways or to precisely define how FA modulates choline metabolism. As a result, while our data suggest a link between FA supplementation and enhanced choline utilization, we cannot establish a direct causal relationship. Additionally, the study did not assess direct markers of insulin signaling, mitochondrial function, fatty acid oxidation, or serum lipoprotein profiles, which limit mechanistic insight into the observed metabolic improvements. Future studies employing isotopic tracer methods, dynamic metabolic testing, and VLDL secretion assays will be essential to clarify how FA influences hepatic lipid metabolism and choline‐dependent processes.

In conclusion, these findings demonstrate that high‐dose FA supplementation reduces hepatic steatosis, as evidenced by lower liver weight and TAG levels, particularly at a tenfold dose. The protective effects are associated with increased hepatic choline concentrations, upregulation of choline‐metabolizing genes, and alterations in the hepatic fatty acid profile suggestive of suppressed de novo lipogenesis. The strong inverse correlation between choline and TAG levels further supports a role for enhanced choline availability in mediating the beneficial effects of FA on liver lipid metabolism. These results highlight the potential metabolic benefits of elevated FA intake in modulating hepatic lipid homeostasis through choline‐dependent pathways.

## Author Contributions

Study conceptualization and design: E.K., R.K., G.H.A.; Sample Collection: E.K., R.K., Z.Y.; Experimental Investigation: E.K., P.V.; Analysis and Data Interpretation: E.K., P.V., R.K., A.H.M., C.T.C., G.H.A.; Provision of Reagents and Instruments: A.H.M., C.T.C., J.L.B., G.H.A.; Funding Acquisition: G.H.A.; Writing – Original Draft: E.K.; Writing – Review and Editing: E.K., R.K., A.H.M., C.T.C., J.L.B., C.E.C., G.H.A.

## Conflicts of Interest

The authors declare no conflicts of interest.

## Supporting information


**Figure S1:** fba270063‐sup‐0001‐FigureS1.docx.

**Figure S2:** fba270063‐sup‐0002‐FigureS2.docx.

**Table S1:** fba270063‐sup‐0003‐TableS1.docx.

**Table S2:** fba270063‐sup‐0004‐TableS2.docx.

**Table S3:** fba270063‐sup‐0005‐TableS3.docx.

**Table S4:** fba270063‐sup‐0006‐TableS4.docx.

**Table S5:** fba270063‐sup‐0007‐TableS5.docx.

## Data Availability

The data that support the findings of this study are available in the Results and Supporting Information sections of this article.
