# Supplementary material for: Folic Acid Supplementation Attenuates Hepatic Steatosis by Enhancing Choline Availability and Remodeling Fatty Acid Profiles in Mice Fed a High‐Fat Diet
Source: FASEB Bioadv. 2025 Oct 29;7(11):e70063. doi: 10.1096/fba.2025-00251 (PMC12569376; doi:10.1096/fba.2025-00251)
Supplement: Supplementary file 1 — Figure S1: fba270063‐sup‐0001‐FigureS1.docx. [file FBA2-7-e70063-s005.docx]

**
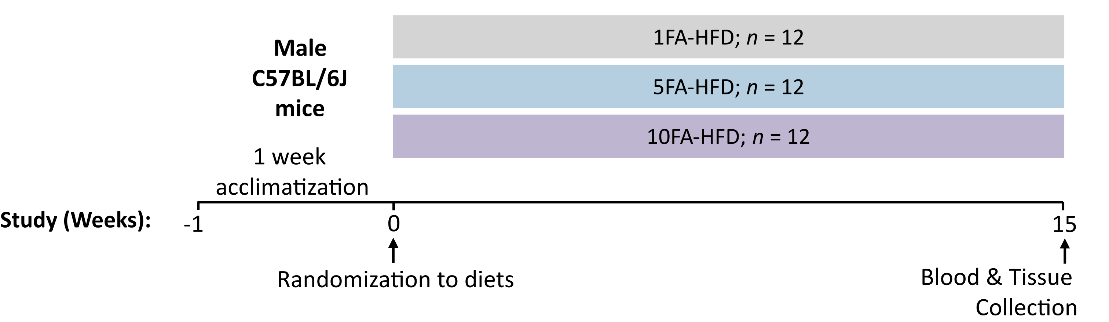
**

**Supplementary Figure 1. Schematic of study design**. Mice were randomized to 45 kcal% fat diets containing folic acid at one-fold, five-fold, or ten-fold AIN-93G recommended amounts with adjustments for energy density. Figure adapted from Kranenburg et al., 2025 (12)
