# Supplementary material for: Folic Acid Supplementation Attenuates Hepatic Steatosis by Enhancing Choline Availability and Remodeling Fatty Acid Profiles in Mice Fed a High‐Fat Diet
Source: FASEB Bioadv. 2025 Oct 29;7(11):e70063. doi: 10.1096/fba.2025-00251 (PMC12569376; doi:10.1096/fba.2025-00251)
Supplement: Supplementary file 2 — Figure S2: fba270063‐sup‐0002‐FigureS2.docx. [file FBA2-7-e70063-s002.docx]

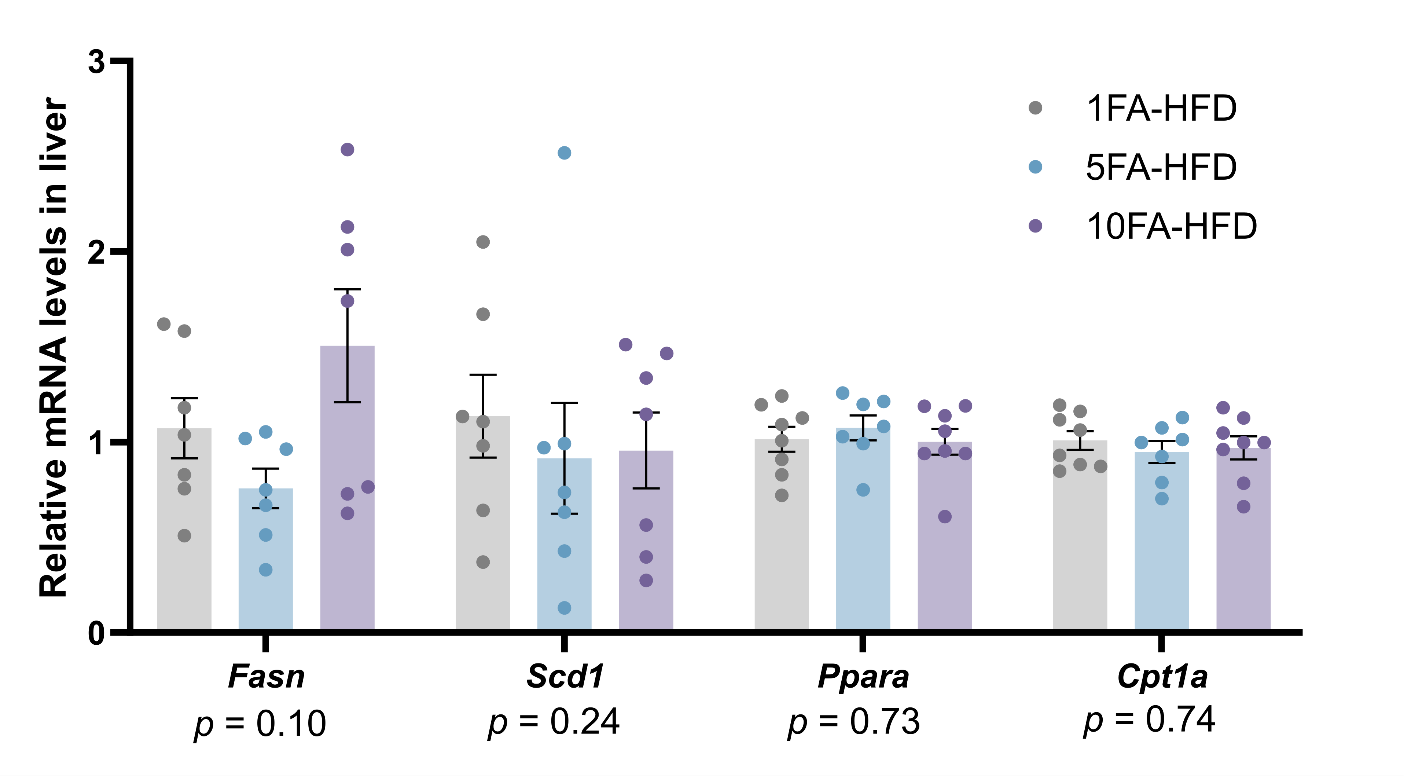


**Supplementary Figure 2. Relative mRNA levels of lipogenic (fatty acid synthase, Fasn, and stearoyl-CoA desaturase-1, *Scd1*) and beta-oxidative (peroxisome proliferator-activated receptor alpha, *Ppara*, and carnitine palmitoyltransferase 1A, *Cpt1a*) genes in the liver**. Data was analysed by one-way ANOVA and presented as means ± S.E.M., *n* = 7-8/group.
