# Supplementary material for: Folic Acid Supplementation Attenuates Hepatic Steatosis by Enhancing Choline Availability and Remodeling Fatty Acid Profiles in Mice Fed a High‐Fat Diet
Source: FASEB Bioadv. 2025 Oct 29;7(11):e70063. doi: 10.1096/fba.2025-00251 (PMC12569376; doi:10.1096/fba.2025-00251)
Supplement: Supplementary file 3 — Table S1: fba270063‐sup‐0003‐TableS1.docx. [file FBA2-7-e70063-s006.docx]

**Supplementary Table 1. Diet composition**

|  | **1FA-HFD** | **5FA-HFD** | **10FA-HFD** |
| --- | --- | --- | --- |
|  | g/kg (kcal%) | | |
| Protein | 203 (20) | 203 (20) | 203 (20) |
| Carbohydrate | 356.3 (35) | 356.3 (35) | 356.3 (35) |
| Fat | 202.5 (45) | 202.5 (45) | 202.5 (45) |
| **Ingredients** | g | | |
| Casein | 200 | 200 | 200 |
| L-cystine | 3 | 3 | 3 |
|  |  |  |  |
| Corn Starch | 72.8 | 72.8 | 72.8 |
| Maltodextrin 10 | 100 | 100 | 100 |
| Sucrose | 172.8 | 172.8 | 172.8 |
|  |  |  |  |
| Cellulose, BW200 | 50 | 50 | 50 |
|  |  |  |  |
| Lard | 177.5 | 177.5 | 177.5 |
| Soybean Oil | 25 | 25 | 25 |
|  |  |  |  |
| t-Butylhydroquinone | 0.014 | 0.014 | 0.014 |
|  |  |  |  |
| Mineral Mix (S10022C, 10X) | 35 | 35 | 35 |
| Vitamin Mix (V10037C, 10X) | 1 | 1 | 1 |
| **Folic Acid, added** | **0** | **0.008** | **0.018** |
|  |  |  |  |
| Choline Bitartrate | 2.5 | 2.5 | 2.5 |
| **Total** | 839.614 | 839.622 | 839.632 |

Table adapted from Kranenburg et al., 2025 (12).
