# Supplementary material for: Folic Acid Supplementation Attenuates Hepatic Steatosis by Enhancing Choline Availability and Remodeling Fatty Acid Profiles in Mice Fed a High‐Fat Diet
Source: FASEB Bioadv. 2025 Oct 29;7(11):e70063. doi: 10.1096/fba.2025-00251 (PMC12569376; doi:10.1096/fba.2025-00251)
Supplement: Supplementary file 4 — Table S2: fba270063‐sup‐0004‐TableS2.docx. [file FBA2-7-e70063-s003.docx]

**Supplementary Table 2. Fatty acid concentrations of hepatic TAGs normalized to total fatty acids.**

| Fatty acids (%) | **1FA-HFD** | **5FA-HFD** | **10FA-HFD** | *p*-value |
| --- | --- | --- | --- | --- |
| **Saturated Fatty Acids** |  |  |  |  |
| Mystiric Acid (C14:0) | 0.49 ± 0.04 | 0.43 ± 0.03 | 0.43 ± 0.04 | 0.342 |
| Palmitic Acid (C16:0) | 22.61 ± 0.56 | 23.10 ± 0.18 | 22.42 ± 0.53 | 0.565 |
| Stearic Acid (C18:0) | 1.89 ± 0.25 | 1.72 ± 0.07 | 1.78 ± 0.10 | 0.695 |
| Arachidic Acid (C20:0) | 0.18 ± 0.01 | 0.22 ± 0.05 | 0.29 ± 0.03 | 0.062 |
| Behenic Acid (C22:0) | 0.02 ± 0.00^a^ | 0.02 ± 0.00^a^ | 0.06 ± 0.02^b^ | 0.015 |
| Σ Saturated Fatty Acids | 25.20 ± 0.35 | 25.50 ± 0.29 | 24.97 ± 0.47 | 0.626 |
| **Monounsaturated Fatty Acids** |  |  |  |  |
| Myristoleic Acid (C14:1) | 0.01 ± 0.00 | 0.01 ± 0.02 | 0.01 ± 0.01 | 0.656 |
| Palmitoleic Acid (C16:1n-7) | 3.39 ± 0.06 | 2.89 ± 0.24 | 2.89 ± 0.34 | 0.276 |
| Oleic Acid (C18:1n-9) | 42.15 ± 0.37 | 39.72 ± 1.01 | 39.50 ± 1.04 | 0.086 |
| Vaccenic Acid (C18:1n-7) | 3.16 ± 0.15 | 2.71 ± 0.30 | 2.65 ± 0.29 | 0.313 |
| Eicosenoic Acid (C20:1n-9) | 1.00 ± 0.06 | 0.86 ± 0.04 | 0.99 ± 0.05 | 0.093 |
| Erucic Acid (C22:1n-9) | 0.05 ± 0.02^a^ | 0.05 ± 0.01^a^ | 0.08 ± 0.01^b^ | 0.007 |
| Σ Monounsaturated Fatty Acids | 49.76 ± 0.43 | 46.24 ± 1.48 | 46.12 ± 1.63 | 0.110 |
| **n-3 Polyunsaturated Fatty Acids** |  |  |  |  |
| Alpha-linolenic Acid (ALA, C18:3n-3) | 0.54 ± 0.06 | 0.66 ± 0.04 | 0.71 ± 0.07 | 0.152 |
| Eicosatrienoic Acid (ETE, C20:3n-3) | 0.04 ± 0.00 | 0.03 ± 0.00 | 0.05 ± 0.00 | 0.058 |
| Eicosapentaenoic Acid (EPA, C20:5n-3) | 0.19 ± 0.02 | 0.30 ± 0.04 | 0.24 ± 0.03 | 0.055 |
| n-3 Docosapentaenoic Acid (DPA, C22:5n-3) | 0.50 ± 0.04 | 0.57 ± 0.04 | 0.55 ± 0.06 | 0.534 |
| Docosahexaenoic Acid (DHA, C22:6n-3) | 1.36 ± 0.11 | 1.55 ± 0.13 | 1.58 ± 0.18 | 0.527 |
| Σ n-3 Polyunsaturated Fatty Acids | 2.63 ±0.15 | 3.11 ± 0.23 | 3.12 ± 0.32 | 0.303 |
| **n-6 Polyunsaturated Fatty Acids** |  |  |  |  |
| Linoleic Acid (C18:2n-6) | 15.00 ± 0.86 | 17.74 ± 1.04 | 17.76 ± 1.43 | 0.176 |
| Gamma Linolenic Acid (C18:3n-6) | 0.29 ± 0.02^ns^ | 0.43 ± 0.05^ns^ | 0.43 ± 0.05^ns^ | 0.049 |
| Eicosadienoic Acid (C20:2n-6) | 0.33 ± 0.04 | 0.29 ± 0.01 | 0.33 ± 0.01 | 0.118 |
| Dihomo-gamma Linolenic Acid (C20:3n-6) | 0.81 ± 0.07 | 0.79 ± 0.04 | 0.82 ± 0.03 | 0.915 |
| Arachidonic Acid (ARA, C20:4n-6) | 1.50 ± 0.09 | 1.80 ± 0.13 | 1.92 ± 0.18 | 0.122 |
| Docosadienoic Acid (C22:2n-6) | 0.40 ± 0.05 | 0.30 ± 0.07 | 0.51 ± 0.34 | 0.326 |
| Adrenic Acid (C22:4n-6) | 0.51 ± 0.04 | 0.46 ± 0.02 | 0.53 ± 0.04 | 0.307 |
| n-6 Docosapentaenoic Acid (C22:5n-6) | 0.21 ± 0.01^a^ | 0.20 ± 0.01^a^ | 0.53 ± 0.03^b^ | 0.020 |
| Σ n-6 Polyunsaturated Fatty Acids | 18.53 ± 0.76 | 21.55 ± 1.20 | 22.05 ± 1.76 | 0.222 |

Different superscript letters indicate statistically significant differences between the means of groups by one-way ANOVA with Tukey-Kramer post-hoc test. Data presented as means ± S.E.M. *n* = 6/group. Abbreviations: 1FA-HFD, one-fold folic acid-high-fat diet; 5FA-HFD, five-fold folic acid-high-fat diet; 10FA-HFD, ten-fold folic acid-high-fat diet; TAGs, triacylglycerol; ns, not significant.
