# Supplementary material for: Folic Acid Supplementation Attenuates Hepatic Steatosis by Enhancing Choline Availability and Remodeling Fatty Acid Profiles in Mice Fed a High‐Fat Diet
Source: FASEB Bioadv. 2025 Oct 29;7(11):e70063. doi: 10.1096/fba.2025-00251 (PMC12569376; doi:10.1096/fba.2025-00251)
Supplement: Supplementary file 5 — Table S3: fba270063‐sup‐0005‐TableS3.docx. [file FBA2-7-e70063-s007.docx]

**Supplementary Table 3. Fatty acid concentrations of PC.**

| Fatty Acids (µmol/g) | 1FA-HFD | 5FA-HFD | 10FA-HFD | *p-value* |
| --- | --- | --- | --- | --- |
| Saturated Fatty Acids |  |  |  |  |
| Lauric Acid (C12:0) | 0.01 ± 0.00 | 0.01 ± 0.00 | 0.01 ± 0.00 | 0.389 |
| Mystiric Acid (C14:0) | 0.05 ± 0.01 | 0.05 ± 0.00 | 0.05 ± 0.01 | 0.926 |
| Pentadecanoic Acid (C15:0) | 0.02 ± 0.00 | 0.02 ± 0.00 | 0.02 ± 0.00 | 0.408 |
| Palmitic Acid (C16:0) | 9.55 ± 0.32 | 9.78 ± 0.30 | 9.49 ± 0.32 | 0.786 |
| Stearic Acid (C18:0) | 5.05 ± 0.20 | 5.15 ± 0.18 | 4.67 ± 0.20 | 0.206 |
| Arachidic Acid (C20:0) | 0.05 ± 0.02 | 0.08 ± 0.01 | 0.07 ± 0.02 | 0.405 |
| Behenic Acid (C22:0) | 0.01 ± 0.01 | 0.02 ± 0.00 | 0.01 ± 0.01 | 0.340 |
| Lignoceric Acid (C24:0) | 0.02 ± 0.00 | 0.02 ± 0.00 | 0.01 ± 0.00 | 0.312 |
| Σ Saturated Fatty Acids | 14.76 ± 0.34 | 15.13 ± 0.31 | 14.34 ± 0.34 | 0.255 |
| Monounsaturated Fatty Acids |  |  |  |  |
| Myristoleic Acid (C14:1) | 0.00 ± 0.01 | 0.02 ± 0.01 | 0.00 ± 0.01 | 0.412 |
| Palmitoleic Acid (C16:1n-7) | 0.21 ± 0.02 | 0.21 ± 0.01 | 0.22 ± 0.02 | 0.772 |
| Sapienic Acid (C16:1n-9) | 0.10 ± 0.01 | 0.09 ± 0.01 | 0.09 ± 0.01 | 0.860 |
| Vaccenic Acid (C18:1n-7) | 0.72 ± 0.05 | 0.68 ± 0.04 | 0.58 ± 0.05 | 0.108 |
| Oleic Acid (C18:1n-9) | 4.24 ± 0.16 | 4.48 ± 0.15 | 4.23 ± 0.16 | 0.426 |
| Gondoic Acid (C20:1n-9) | 0.08 ± 0.01 | 0.08 ± 0.01 | 0.08 ± 0.01 | 0.552 |
| Erucic Acid (C22:1n-9) | 0.04 ± 0.01 | 0.06 ± 0.01 | 0.05 ± 0.01 | 0.095 |
| Nervonic Acid (C24:1n-9) | 0.01 ± 0.00 | 0.01 ± 0.00 | 0.01 ± 0.00 | 0.365 |
| Σ Monounsaturated Fatty Acids | 5.40 ± 0.19 | 5.63 ± 0.18 | 5.26 ± 0.19 | 0.359 |
| n-3 Polyunsaturated Fatty Acids |  |  |  |  |
| α-linolenic Acid (ALA, C18:3n-3) | 0.02 ± 0.00 | 0.02 ± 0.00 | 0.03 ± 0.00 | 0.264 |
| Eicosatrienoic Acid (ETE, C20:3n-3) | 0.02 ± 0.00 | 0.02 ± 0.00 | 0.01 ± 0.00 | 0.416 |
| Eicosapentaenoic Acid (EPA, C20:5n-3) | 0.05 ± 0.01 | 0.05 ± 0.01 | 0.05 ± 0.01 | 0.954 |
| n-3 Docosapentaenoic Acid (DPA, C22:5n-3) | 0.11 ± 0.01 | 0.15 ± 0.01 | 0.12 ± 0.01 | 0.104 |
| Docosahexaenoic Acid (DHA, C22:6n-3) | 4.80 ± 0.55 | 5.85 ± 0.51 | 4.86 ± 0.55 | 0.299 |
| Σ n-3 polyunsaturated fatty acids | 4.99 ± 0.56 | 6.09 ± 0.52 | 5.07 ± 0.56 | 0.295 |
| n-6 Polyunsaturated Fatty Acids |  |  |  |  |
| Linoleic Acid (C18:2n-6) | 3.90 ± 0.29 | 4.44 ± 0.27 | 4.85 ± 0.29 | 0.103 |
| γ-Linolenic Acid (C18:3n-6) | 0.05 ± 0.02 | 0.10 ± 0.02 | 0.08 ± 0.02 | 0.247 |
| Eicosadienoic Acid (C20:2n-6) | 0.12 ± 0.01 | 0.12 ± 0.01 | 0.12 ± 0.01 | 0.967 |
| Dihomo-γ-Linolenic Acid (C20:3n-6) | 1.00 ± 0.08 | 0.96 ± 0.08 | 0.82 ± 0.08 | 0.295 |
| Arachidonic Acid (ARA C20:4n-6) | 6.44 ± 0.45 | 7.36 ± 0.41 | 6.20 ± 0.45 | 0.157 |
| Adrenic Acid (C22:4n-6) | 0.92 ± 0.51 | 0.06 ± 0.51 | 0.06 ± 0.55 | 0.420 |
| n-6 Docosapentaenoic Acid (C22:5n-6) | 0.13 ± 0.02 | 0.14 ± 0.02 | 0.15 ± 0.02 | 0.689 |
| Σ n-6 polyunsaturated fatty acids | 11.70 ± 0.61 | 13.19 ± 0.57 | 12.28 ± 0.61 | 0.226 |

Different superscript letters indicate statistically significant differences between the means of groups by one-way ANOVA with Tukey-Kramer post-hoc test. Data presented as means ± S.E.M. *n* = 6-7/group. Abbreviations: 1FA-HFD, one-fold folic acid-high-fat diet; 5FA-HFD, five-fold folic acid-high-fat diet; 10FA-HFD, ten-fold folic acid-high-fat diet; PC, phosphatidylcholine.
