# Supplementary material for: Folic Acid Supplementation Attenuates Hepatic Steatosis by Enhancing Choline Availability and Remodeling Fatty Acid Profiles in Mice Fed a High‐Fat Diet
Source: FASEB Bioadv. 2025 Oct 29;7(11):e70063. doi: 10.1096/fba.2025-00251 (PMC12569376; doi:10.1096/fba.2025-00251)
Supplement: Supplementary file 6 — Table S4: fba270063‐sup‐0006‐TableS4.docx. [file FBA2-7-e70063-s004.docx]

**Supplementary Table 4. Fatty acid concentrations of hepatic PC normalized to total fatty acids.**

| Fatty Acid (%) | 1FA-HFD | 5FA-HFD | 10FA-HFD | *p-value* |
| --- | --- | --- | --- | --- |
| Saturated Fatty Acids |  |  |  |  |
| Lauric Acid (C12:0) | 0.02 ± 0.00 | 0.02 ± 0.00 | 0.02 ± 0.00 | 0.447 |
| Mystiric Acid (C14:0) | 0.12 ± 0.01 | 0.11 ± 0.01 | 0.12 ± 0.01 | 0.781 |
| Pentadecanoic Acid (C15:0) | 0.05 ± 0.00 | 0.05 ± 0.00 | 0.05 ± 0.00 | 0.598 |
| Palmitic Acid (C16:0) | 24.40 ± 1.08 | 23.16 ± 1.00 | 24.18 ± 1.08 | 0.668 |
| Stearic Acid (C18:0) | 14.30 ± 0.44 | 13.42 ± 0.40 | 13.17 ± 0.44 | 0.186 |
| Arachidic Acid (C20:0) | 0.16 ± 0.04 | 0.23 ± 0.04 | 0.22 ± 0.04 | 0.453 |
| Behenic Acid (C22:0) | 0.05 ± 0.01 | 0.06 ± 0.01 | 0.04 ± 0.01 | 0.432 |
| Lignoceric Acid (C24:0) | 0.06 ± 0.01 | 0.06 ± 0.01 | 0.05 ± 0.01 | 0.427 |
| Σ Saturated Fatty Acids | 39.15 ± 1.15 | 37.12 ± 1.07 | 37.84 ± 1.15 | 0.446 |
| Monounsaturated Fatty Acids |  |  |  |  |
| Myristoleic Acid (C14:1) | 0.01 ± 0.02 | 0.03 ± 0.01 | 0.01 ± 0.02 | 0.431 |
| Palmitoleic Acid (C16:1n-7) | 0.52 ± 0.04 | 0.49 ± 0.04 | 0.56 ± 0.04 | 0.528 |
| Sapienic Acid (C16:1n-9) | 0.24 ± 0.02 | 0.22 ± 0.02 | 0.23 ± 0.02 | 0.752 |
| Vaccenic Acid (C18:1n-7) | 2.03 ± 0.12 | 1.78 ± 0.11 | 1.64 ± 0.12 | 0.095 |
| Oleic Acid (C18:1n-9) | 11.92 ± 0.38 | 11.63 ± 0.35 | 11.85 ± 0.38 | 0.839 |
| Gondoic Acid (C20:1n-9) | 0.25 ± 0.01 | 0.24 ± 0.01 | 0.23 ± 0.01 | 0.508 |
| Erucic Acid (C22:1n-9) | 0.14 ± 0.01 | 0.18 ± 0.01 | 0.15 ± 0.01 | 0.205 |
| Nervonic Acid (C24:1n-9) | 0.02 ± 0.01 | 0.03 ± 0.01 | 0.02 ± 0.01 | 0.392 |
| Σ Monounsaturated Fatty Acids | 15.13 ± 0.47 | 14.59 ± 0.44 | 14.68 ± 0.47 | 0.677 |
| n-3 Polyunsaturated Fatty Acids |  |  |  |  |
| α-linolenic Acid (ALA, C18:3n-3) | 0.05 ± 0.01 | 0.06 ± 0.01 | 0.07 ± 0.01 | 0.220 |
| Eicosatrienoic Acid (ETE, C20:3n-3) | 0.05 ± 0.00 | 0.05 ± 0.00 | 0.04 ± 0.00 | 0.612 |
| Eicosapentaenoic Acid (EPA, C20:5n-3) | 0.14 ± 0.01 | 0.13 ± 0.01 | 0.14 ± 0.01 | 0.693 |
| n-3 Docosapentaenoic Acid (DPA, C22:5n-3) | 0.37 ± 0.03 | 0.44 ± 0.03 | 0.39 ± 0.03 | 0.127 |
| Docosahexaenoic Acid (DHA, C22:6n-3) | 10.72 ± 0.75 | 11.93 ± 0.70 | 10.89 ± 0.75 | 0.455 |
| Σ n-3 Polyunsaturated Fatty Acids | 11.32 ± 0.78 | 12.61 ± 0.72 | 11.53 ± 0.78 | 0.442 |
| n-6 Polyunsaturated Fatty Acids |  |  |  |  |
| Linoleic Acid (C18:2n-6) | 10.80 ± 0.58^a^ | 11.43 ± 0.54^a^ | 14.16 ± 0.64^b^ | 0.004 |
| γ-Linolenic Acid (C18:3n-6) | 0.14 ± 0.05 | 0.25 ± 0.05 | 0.22 ± 0.05 | 0.275 |
| Eicosadienoic Acid (C20:2n-6) | 0.37 ± 0.02 | 0.35 ± 0.02 | 0.37 ± 0.02 | 0.514 |
| Dihomo-γ-Linolenic Acid (C20:3n-6) | 3.03 ± 0.21 | 2.67 ± 0.19 | 2.50 ± 0.21 | 0.202 |
| Arachidonic Acid (ARA C20:4n-6) | 19.44 ± 0.82 | 20.36 ± 0.76 | 18.72 ± 0.82 | 0.355 |
| Adrenic Acid (C22:4n-6) | 0.19 ± 0.01 | 0.19 ± 0.01 | 0.19 ± 0.01 | 0.954 |
| n-6 Docosapentaenoic Acid (C22:5n-6) | 0.43 ± 0.05 | 0.43 ± 0.05 | 0.50 ± 0.05 | 0.597 |
| Σ n-6 Polyunsaturated Fatty Acids | 34.40 ± 0.68 | 35.69 ± 0.63 | 35.95 ± 0.68 | 0.254 |

Different superscript letters indicate statistically significant differences between the means of groups by one-way ANOVA with Tukey-Kramer post-hoc test. Data presented as means ± S.E.M. *n* = 6-7/group. Abbreviations: 1FA-HFD, one-fold folic acid-high-fat diet; 5FA-HFD, five-fold folic acid-high-fat diet; 10FA-HFD, ten-fold folic acid-high-fat diet; PC, phosphatidylcholine.
