# Supplementary material for: Folic Acid Supplementation Attenuates Hepatic Steatosis by Enhancing Choline Availability and Remodeling Fatty Acid Profiles in Mice Fed a High‐Fat Diet
Source: FASEB Bioadv. 2025 Oct 29;7(11):e70063. doi: 10.1096/fba.2025-00251 (PMC12569376; doi:10.1096/fba.2025-00251)
Supplement: Supplementary file 7 — Table S5: fba270063‐sup‐0007‐TableS5.docx. [file FBA2-7-e70063-s001.docx]

**Supplementary Table 5. Fatty acid concentrations of hepatic PE normalized to total fatty acids.**

| Fatty Acid (%) | 1FA-HFD | 5FA-HFD | 10FA-HFD | *p-value* |
| --- | --- | --- | --- | --- |
| Saturated Fatty Acids |  |  |  |  |
| Lauric Acid (C12:0) | 0.03 ± 0.01 | 0.01 ± 0.01 | 0.03 ± 0.01 | 0.076 |
| Mystiric Acid (C14:0) | 0.15 ± 0.02 | 0.10 ± 0.02 | 0.19 ± 0.02 | 0.083 |
| Pentadecanoic Acid (C15:0) | 0.04 ± 0.00^ab^ | 0.04 ± 0.00^a^ | 0.04 ± 0.00^b^ | 0.037 |
| Palmitic Acid (C16:0) | 18.54 ± 0.70 | 16.66 ± 0.65 | 18.60 ± 0.70 | 0.093 |
| Stearic Acid (C18:0) | 18.59 ± 0.34 | 18.27 ± 0.31 | 18.67 ± 0.34 | 0.664 |
| Arachidic Acid (C20:0) | 0.20 ± 0.02 | 0.20 ± 0.02 | 0.26 ± 0.02 | 0.051 |
| Behenic Acid (C22:0) | 0.06 ± 0.00 | 0.05 ± 0.00 | 0.06 ± 0.00 | 0.137 |
| Lignoceric Acid (C24:0) | 0.08 ± 0.01 | 0.08 ± 0.01 | 0.09 ± 0.01 | 0.345 |
| Σ Saturated Fatty Acids | 37.67 ± 0.95 | 35.43 ± 0.88 | 37.95 ± 0.95 | 0.128 |
| Monounsaturated Fatty Acids |  |  |  |  |
| Myristoleic Acid (C14:1) | 0.01 ± 0.01 | 0.01 ± 0.01 | 0.02 ± 0.01 | 0.456 |
| Palmitoleic Acid (C16:1n-7) | 0.29 ± 0.02 | 0.26 ± 0.02 | 0.31 ± 0.02 | 0.184 |
| Sapienic Acid (C16:1n-9) | 0.04 ± 0.01 | 0.03 ± 0.01 | 0.04 ± 0.01 | 0.346 |
| Vaccenic Acid (C18:1n-7) | 1.30 ± 0.08 | 1.13 ± 0.07 | 1.09 ± 0.08 | 0.144 |
| Oleic Acid (C18:1n-9) | 14.71 ± 0.36^a^ | 13.13 ± 0.33^b^ | 14.19 ± 0.39^ab^ | 0.016 |
| Gondoic Acid (C20:1n-9) | 0.32 ± 0.01 | 0.30 ± 0.01 | 0.32 ± 0.01 | 0.425 |
| Erucic Acid (C22:1n-9) | 0.27 ± 0.02 | 0.26 ± 0.02 | 0.29 ± 0.02 | 0.709 |
| Nervonic Acid (C24:1n-9) | 0.03 ± 0.00 | 0.04 ± 0.00 | 0.04 ± 0.00 | 0.580 |
| Σ Monounsaturated Fatty Acids | 16.98 ± 0.45^a^ | 15.17 ± 0.42^b^ | 16.53 ± 0.45^ab^ | 0.023 |
| n-3 Polyunsaturated Fatty Acids |  |  |  |  |
| α-linolenic Acid (ALA, C18:3n-3) | 0.05 ± 0.01 | 0.05 ± 0.01 | 0.06 ± 0.01 | 0.421 |
| Eicosatrienoic Acid (ETE, C20:3n-3) | 0.03 ± 0.00 | 0.03 ± 0.00 | 0.03 ± 0.00 | 0.215 |
| Eicosapentaenoic Acid (EPA, C20:5n-3) | 0.16 ± 0.01 | 0.18 ± 0.01 | 0.15 ± 0.01 | 0.301 |
| n-3 Docosapentaenoic Acid (DPA, C22:5n-3) | 0.49 ± 0.03 | 0.60 ± 0.03 | 0.54 ± 0.03 | 0.085 |
| Docosahexaenoic Acid (DHA, C22:6n-3) | 16.49 ± 0.78^a^ | 19.59 ± 0.72^b^ | 15.93 ± 0.78^a^ | 0.007 |
| Σ n-3 Polyunsaturated Fatty Acids | 17.22 ± 0.81^a^ | 20.45 ± 0.75^b^ | 16.71 ± 0.81^a^ | 0.007 |
| n-6 Polyunsaturated Fatty Acids |  |  |  |  |
| Linoleic Acid (C18:2n-6) | 4.16 ± 0.16^a^ | 4.13 ± 0.14^a^ | 5.00 ± 0.17^b^ | 0.003 |
| γ-Linolenic Acid (C18:3n-6) | 0.02 ± 0.01 | 0.02 ± 0.01 | 0.03 ± 0.01 | 0.908 |
| Eicosadienoic Acid (C20:2n-6) | 0.15 ± 0.01 | 0.15 ± 0.01 | 0.16 ± 0.01 | 0.364 |
| Dihomo-γ-Linolenic Acid (C20:3n-6) | 0.67 ± 0.04 | 0.68 ± 0.03 | 0.61 ± 0.04 | 0.318 |
| Arachidonic Acid (ARA C20:4n-6) | 22.13 ± 0.40 | 22.96 ± 0.37 | 22.09 ± 0.40 | 0.217 |
| Adrenic Acid (C22:4n-6) | 0.48 ± 0.03 | 0.47 ± 0.03 | 0.49 ± 0.03 | 0.906 |
| n-6 Docosapentaenoic Acid (C22:5n-6) | 0.53 ± 0.07 | 0.55 ± 0.06 | 0.63 ± 0.07 | 0.605 |
| Σ n-6 Polyunsaturated Fatty Acids | 28.13 ± 0.40 | 28.95 ± 0.37 | 28.81 ± 0.40 | 0.312 |

Different superscript letters indicate statistically significant differences between the means of groups by one-way ANOVA with Tukey-Kramer post-hoc test. Data presented as means ± S.E.M. *n* = 6-7/group. Abbreviations: 1FA-HFD, one-fold folic acid-high-fat diet; 5FA-HFD, five-fold folic acid-high-fat diet; 10FA-HFD, ten-fold folic acid-high-fat diet; PE, phosphatidylethanolamine.
